# An *In-Silico* Study on the Most Effective Growth Factors in Retinal Regeneration Utilizing Tissue Engineering Concepts

**DOI:** 10.18502/jovr.v16i1.8251

**Published:** 2021-01-20

**Authors:** Nima Beheshtizadeh, Alireza Baradaran-Rafii, Maryam Sharifi Sistani, Mahmoud Azami

**Affiliations:** ^1^Department of Tissue Engineering and Applied Cell Sciences, School of Advanced Technologies in Medicine, Tehran University of Medical Sciences, Tehran, Iran; ^2^Students’ Scientific Research Center, Tehran University of Medical Sciences, Tehran, Iran; ^3^Ocular Tissue Engineering Research Center, Research Institute for Ophthalmology and Vision Science, Shahid Beheshti University of Medical Sciences, Tehran, Iran

**Keywords:** Effective Growth Factors, In-silico Study, Regenerative Medicine, Retinal Tissue Engineering, Systems Biology

## Abstract

**Purpose:**

Considering the significance of retinal disorders and the growing need to employ tissue engineering in this field, *in-silico* studies can be used to establish a cost-effective method. This *in-silico* study was performed to find the most effective growth factors contributing to retinal tissue engineering.

**Methods:**

In this study, a regeneration gene database was used. All 21 protein-coding genes participating in retinal regeneration were considered as a protein–protein interaction (PPI) network via the “STRING App” in “Cytoscape 3.7.2” software. The resultant graph possessed 21 nodes as well as 37 edges. Gene ontology (GO) analysis, as well as the centrality analysis, revealed the most effective proteins in retinal regeneration.

**Results:**

According to the biological processes and the role of each protein in different pathways, selecting the correct one is possible through the information that the network provides. Eye development, detection of the visible light, visual perception, photoreceptor cell differentiation, camera-type eye development, eye morphogenesis, and angiogenesis are the major biological processes in retinal regeneration. Based on the GO analysis, SHH, STAT3, FGFR1, OPN4, ITGAV, RAX, and RPE65 are effective in retinal regeneration via the biological processes. In addition, based on the centrality analysis, four proteins have the greatest influence on retinal regeneration: SHH, IGF1, STAT3, and ASCL1.

**Conclusion:**

With the intention of applying the most impressive growth factors in retinal engineering, it seems logical to pay attention to SHH, STAT3, and RPE65. Utilizing these proteins can lead to fabricate high efficiency engineered retina via all aforementioned biological processes.

##  INTRODUCTION

The aim of regenerative medicine as the primary process involved in cell growth and organ reconstruction is to return the main cell functions and recovery of the damaged tissue or organ via its replacing or regenerating.^[1]^ In fact, there are three solutions for patients having organ impairment based on the severity of the destruction: graft implantation, substitution, and restoration. Graft implantation has an extensive waiting list candidates all around the world; for example, the organ transplantation waiting list is updated every 15 min in the United States of America.^[2]^ The ultimate prospect of tissue engineering is creating and providing tissues that are preferably autologous in organ substitutions through cells and biomaterials utilization simultaneously.^[3,4]^ Besides, tissue engineering has been determined as an efficient method to assist in rescuing lives and improving the quality of life.

Considering the major components for tissue engineering, that is, scaffolds, cells, and growth factors and a variety of their available options would highlight the fact that selecting the most appropriate ones to fabricate an engineered tissue demands an optimization system. In fact, a wide range of biomaterials can be used as scaffolds; polymers and hydrogels are the most commonly used materials in this field.^[5,6,7]^ Selecting the appropriate material is in close relation with the destination tissue. Poly-lactide-co-glycolide (PLGA), poly-caprolactone (PCL), poly-glycerol sebacate (PGS), and polymethyl methacrylate (PMMA) are some of the high consumption polymers in retinal tissue engineering.

In addition to scaffolds, growth factors play an essential role in tissue engineering.^[8]^ Growth factors are generally the regulators of substances, namely proteins or hormones that can stimulate cell proliferation and differentiation. Growth factors play an important role in the healing and regeneration of the retina. Retinal disorders directly affect vision; therefore, retinal tissue engineering is fundamental.^[3,9,10,11]^ To understand the effective mechanisms in this process, it is better to compare growth factors' interaction with each other and then select the most appropriate one.

Looking at the literature, retinal regeneration and retinal tissue engineering have been studied by several researchers.^[12,13,14,15,16,17,18,19,20,21]^ Liu *et al* ^[22]^ studied the application of hyaluronic acid (HA) hydrogels in retinal progenitor cell transplantation. Their reason for selecting HA was its role as a feeder layer in stem cell cultures. In addition, the relative ease with which various parameters could be controlled (e.g., hydrogel architecture, mechanics, and degradation) was effective in choosing the HA hydrogel. They concluded that HA hydrogels, with their developmentally relevant composition and malleable physical properties, provide a unique microenvironment for self-renewal and differentiation of the retinal progenitor cells (RPCs) for retinal repair. Furthermore, Fausett *et al* ^[23]^ showed that in the damaged zebrafish retina, the Muller glia re-enter the cell cycle, increase α1tubulin (α1T) promoter activity, and generate new neurons and glia for retinal repair. They suggested that the achaete-scute family bHLH transcription factor 1a (ASCL1a) is required to convert the quiescent Muller glia into the actively dividing retinal progenitors, and that ASCL1a is a key regulator in initiating the retinal regeneration.

Kador and Goldberg^[24]^ studied the delivery of cell transplants for retinal degeneration. Focusing on the photoreceptor and progenitor-directed approaches, the authors reviewed how advances in tissue engineering and cell scaffold design were enhancing cell therapies for retinal degeneration. Furthermore, Yao *et al* ^[25]^ reviewed the current literature on synthetic polymer scaffolds used for stem cell transplantation, especially RPCs. The advantages and disadvantages of different polymer scaffolds, the role of different surface modifications on cell attachment and differentiation, and the controlled drug delivery were discussed in their paper. Tao and Klassen^[18]^ have also presented a wide range of practical biomaterials in retinal tissue engineering. They studied the role of stem cells in retinal repair, and then focused on the material side, followed by considering cells and materials in combination. They also examined the current status of retinal tissue engineering and looked ahead to the challenges that investigators are involved within this field. In addition, Bainbridge *et al* ^[26]^ published their preliminary results of gene therapy for retinal degeneration. In their study, the patients were enrolled in trials of recombinant adeno-associated viral delivery of the retinoid isomerohydrolase (RPE65), which was administered as a subretinal injection during vitrectomy. The preliminary results from their investigations suggested that the procedure was safe in the short term, and their data were suggestive of efficacy.

Furthermore, Nelson *et al* ^[27]^ found out that signal transducer and activator of transcription 3 (STAT3) expression was observed in all Muller glia, whereas ASCL1a expression was restricted to only the mitotic ones. They suggested that while ASCL1a and Lin-28 homolog A (LIN28a) are required for Muller glia proliferation, STAT3 is necessary for the maximal number of Muller glia to proliferate during regeneration of the damaged zebrafish retina. In another study, Spence *et al* ^[28]^ worked on the fibroblast growth factor (FGF)–hedgehog (SHH) interdependence during retinal regeneration. Their results support a model where the FGF and SHH pathways work together to stimulate retinal regeneration.

Recently, Singh *et al* ^[29]^ reviewed retinal tissue engineering from the pluripotent stem cells and summarized the progress in cell therapies of the retina, with a focus on the human pluripotent stem cell-derived retinal tissue, and critically evaluated the potential of retinal organoid approaches to solve a major unmet clinically needed retinal repair and vision restoration in conditions caused by retinal degeneration and traumatic ocular injuries.

Based on the published works, it can be concluded that there is no comprehensive study on the retinal growth factors that can draw up the existing relation among them. In addition, to the best of our knowledge, there is no *in-silico* study of retinal tissue engineering. In fact, in retinal regeneration, several proteins are used therapeutically. If the interaction between them would be clear, and the biological function of each one is determined, they can be used as growth factors in retinal tissue engineering.

In order to get the best results from the *in-vitro* and *in-vivo* tests, it is needed to select the best growth factors based on previous experiments and existing data. However, there are many reports about the effects of using each growth factor without any coherence and correlation among them. It seems that describing the interactions among growth factors is a critical fact that would lighten up the retinal tissue engineering path, that is, possible effects of increasing the amount of a growth factor on other growth factors' functions. One of the least expensive methods for detecting this kind of facts is evaluating them with an *in-silico* study.

In this work, retinal growth factors interactions have been studied via creating their interaction network. By creating this network, the influence of each growth factor on the biological processes can be determined. The higher degree in this network leads to higher interactions among them and causes much more effect. The main goal of this study is to find out which kind of retinal growth factor should be used to have the highest effect on the desired biological process.

##  METHODS

All *in-vivo* or *in-vitro *studies already performed on retinal tissue engineering were reviewed to know how cells were affected by their surrounding environmental factors. The final results of these studies were collected into databases to provide access to comprehensive and accurate information. In the current study, the regeneration gene database was used.^[30]^ According to this database, 21 protein-coding genes participate in retinal regeneration. In order to reveal their interaction and realize how they affect each other, all of these proteins were gathered from this database. Then a study on systems biology was performed. The mathematical modelling of complicated biological systems is called systems biology. For this purpose, the “STRING App” in “Cytoscape 3.7.2 software” ^[1]^ was utilized. The STRING App is one of the Cytoscape software apps related to the STRING database.^[31]^ This database is utilized for investigating the protein–protein interaction (PPI).

In this regard, the data source was adjusted on “STRING: protein query”, and all 21 proteins were included in this query. Given that this study is on human proteins, the species section was set on *Homo sapiens*, and the analysis results were matched for humans. Selecting default options from multiple possible matches found for some proteins would lead to loading interactions from the STRING database. Then, a primitive model of PPI graph was drawn, meanwhile having the ability to alter into other layouts, that is, grid, circular, and hierarchical.

Creating a PPI network, there are 21 nodes and 37 edges. In other words, a 21 node-included graph was drawn using the STRING App. Then, gene ontology (GO) analysis was performed. GO analysis is a major bioinformatics initiative to unify the representation of gene and gene product attributes across all species.^[32]^ The protein–coding genes which are involved in retinal regeneration are listed in Table 1.

**Table 1:** The list of protein-coding genes that are involved in retinal regeneration


**No**	**Name**	**Gene ID**	**Degree**
1	SHH	6469	10
2	IGF1	3479	8
3	STAT3	6774	8
4	ASCL1	429	7
5	CDH2	1000	7
6	WNT3A	89780	5
7	FGFR1	2260	5
8	VTN	7448	5
9	CALB2	794	3
10	CNTF	1270	3
11	MDK	4192	2
12	INSM1	3642	2
13	OPN4	94233	2
14	ITGAV	3685	2
15	C3	718	2
16	RAX	30062	1
17	RPE65	6121	1
18	APOBEC2	10930	0
19	TUBA1C	84790	0
20	HSPA1L	3305	0
21	VPS35	55737	0

In addition, a centrality analysis was performed based on the degree index. In network analysis, based on graph theory, centrality indicators identify the most important nodes within a network. The results of this analysis lead to a degree based array of nodes in the network. In order to illustrate comprehensible figures, the circular layout was selected. Also, to recognize the most effective proteins, degree sorted layouts were selected.

##  RESULTS 

**Figure 1:**
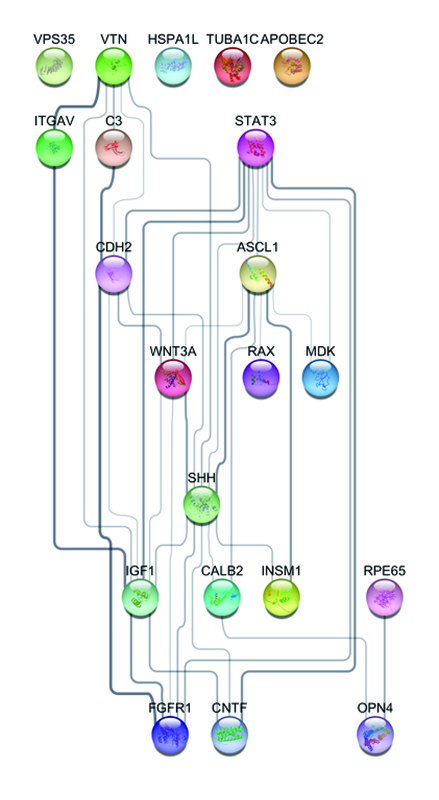
Hierarchical layout of the PPI network performed by STRING App in Cytoscape 3.7.2. This network presents GO analysis of retina regeneration effective growth. Each circle provides a schematic drawing of protein structure. The colors are set randomly, and the connection line's thickness illustrates the relation of power. Also, a thicker line presents much more evidence and documents to approve the connectivity.

Figure 1 shows the PPI network. In this figure, a PPI network and a GO analysis of retinal regeneration effective growth factors presented by STRING App database are shown. This figure presents the relationship among all proteins participating in retinal regeneration. These proteins are also listed in Table 1.

Based on Figure 1, there are four proteins that act individually: heat shock protein family A member 1 (HSPA1L), VPS35 retromer complex component (VPS35), apolipoprotein B mRNA editing enzyme catalytic subunit 2 (APOBEC2), and tubulin alpha 1c (TUBA1C). These proteins do not have any interactions with the other 17 effective proteins in the retina healing process. Mentioned proteins can show activity individually or via activating other proteins. For instance, the VPS35 impression is on the upregulation of the development process. As a matter of fact, there are 11 proteins involved in the upregulation of the development process, and VPS35 is one of them.

**Figure 2:**
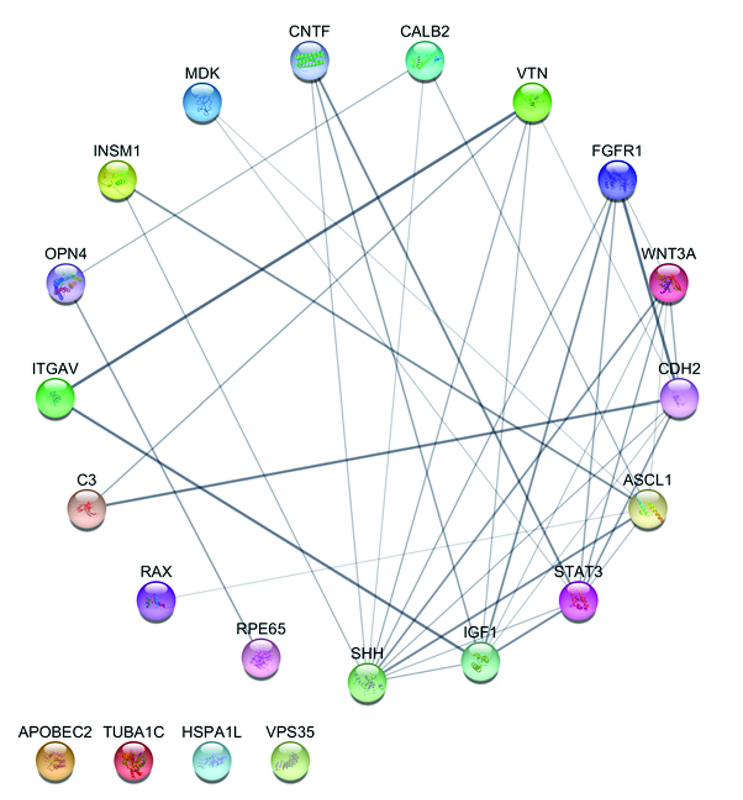
The degree-sorted circular layout of the PPI network performed by STRING App in Cytoscape 3.7.2. Each circle provides a schematic drawing of protein structure. The colors are set randomly, and the connection line's thickness illustrates the relation of power. Also, a thicker line presents much more evidence and documents to approve the connectivity.

As mentioned previously, a centrality analysis based on the degree was performed. In order to get the best understanding, it is preferred to present the PPI network by the centrality analysis based on the degree. Figure 2 illustrates this analysis.

According to this centrality analysis, four proteins have the greatest influence on retinal regeneration: SHH, insulin-like growth factor 1 (IGF1), STAT3, and achaete-scute family bHLH transcription factor 1 (ASCL1). These proteins have the highest degree in the PPI network.

The most impressive biological processes considered in this study, that is, eye development, detection of visible lights, visual perception, photoreceptor cell differentiation, camera-type eye development, eye morphogenesis, and angiogenesis, lead to retinal regeneration. Moreover, based on GO analysis, the most effective protein-coding genes that act in the mentioned biological procedures are SHH, STAT3, FGFR1, Opsin 4 (OPN4), integrin subunit alpha V (ITGAV), retina and anterior neural fold homeobox (RAX), and RPE65.

##  DISCUSSION

In this study, two analyses were performed: GO analysis and centrality analysis. Based on the GO analysis results, there were seven proteins participating in seven biological processes. In addition, the four most effective proteins in the retinal regeneration process were identified via degree index-based centrality analysis. To get the most appropriate growth factor for use in retinal tissue engineering, each protein's role needs to be identified.

Considering the four isolated proteins in Figure 1, it can be argued that these proteins are also involved in retinal regeneration; however, there should be some biological processes that these proteins could impress on. Positive regulation of transport is a biological process in which VPS35 and HSPA1L participate. Based on GO analysis (GO: 0051094), a process that causes and expands the rate of development is an “upregulation of developmental process”; it points to a biological procedure, which results in the development of an organism from the primary situation till the last condition; for example, from a zygote to an adult. In addition, “Transport positive upregulation” is a process that grows the scope, rate, and frequency of substances movement such as ions, molecules in cells and between them by use of a factor, for example, a pore or a transporter (GO: 0051050).^[33]^

The four aforementioned proteins, which have the most influence on retinal regeneration based on centrality analysis, are SHH, IGF1, STAT3, and ASCL1. SHH is a protein functional in embryo formation. Interestingly, SHH and FGF can induce stem/progenitor cells in the regeneration process, and these two have simultaneous interdependence on each other. For example, if SHH is inhibited, FGF would also be inhibited and vice versa. Therefore, FGF and Hedgehog pathways work together to stimulate retinal regeneration. In fact, the complex relation between SHH and FGF regulates this process.^[18]^ SHH has the highest degree in the network (Figure 2). It could be noted that the Hedgehog pathway plays an essential role as a modulator of retinal regeneration.^[28]^

IGF-1 has an impact on the activity of growth promotion. Nerve injury causes phospho-Akt inactivation; therefore, retinal ganglion cell (RGC) loss would occur. It is evident from the literature that supplementation of IGF-1-induced phospho-Akt expression upregulates and provides the cell survival of RGCs.^[34]^ Consequently, during primary levels of nerve damage, IGF-1 would be a key molecule that possesses the apoptosis effect on RGCs.^[34]^ IGF-1 is at the second rank according to its special role in glial cell survival.

Moreover, STAT3 is a transcription factor involved in retinal regeneration, supporting stem cell maintenance and tissue development. Furthermore, Muller glial cells are the kind of cells in the retina that support neurons like other glial cells. The role of STAT3 in the regeneration process is providing maximum proliferation of Muller glia cells in retinal damage.

In fact, STAT3 and ASCL1 have an important place in retinal regeneration. Considering and investing on them in tissue fabrication seems logical to get closer to the regeneration purpose. The study provides a mutual relation between the ASCL1 factor and STAT3 in the regeneration process. STAT3 is expressed in all Muller glial cells, while ASCL1 is only expressed in proliferating Muller glial cells. Although the expression of the ASCL1 is necessary for retinal regeneration, STAT3 in cell proliferation has priority to ASCL1.

Moreover, ASCL1 takes part in STAT3 expression. Both factors are efficient in the regeneration of cell cycles. ASCL1 is a critical regulatory factor in retinal regeneration. It helps and converts dormant Muller glia to retinal progenitors that are able to divide. ASCL1 is a protein expressed during retinal puncture and causes retinal regeneration by affecting the LIN-28 factor.^[23,27]^

Hence, according to centrality analysis, ASCL1 is in relation with two important and high degree factors of the network, SHH and STAT3. As mentioned before, the relation of ASCL1 and STAT3 is direct and mutual, so it is important to consider the role of ASCL1 in glial cell proliferation and survival, which STAT3 is also involved in.

The role of these four proteins is critical in retinal regeneration. Furthermore, GO analysis demonstrated that these proteins have incredible effects on some biological processes. Seven most important biological processes were studied in this work. Table 2 shows the biological processes and the relation with the mentioned protein-coding genes.

**Table 2:** Proteins involved in Retina Biological Processes


**Biological process/ Protein names**	**SHH**	**STAT3**	**FGFR1**	**OPN4**	**ITGAV**	**RAX**	**RPE65**
Eye development	*	*		*	*
Detection of visible lights		*		*
Visual perception		*	*	*
Photoreceptor cell differentiation	*			*
Camera-type eye development	*			*	*
Eye morphogenesis	*			*
Angiogenesis	*	*	*	

Based on GO Analysis, “eye development” (GO: 0001654) is a process which its significant result is eye development over time along with the formation of the matured structures. In addition, “detection of visible lights” is a chain of incidents that a visible light stimulus is captured by a cell and turned into a molecular signal (GO: 0009584). “Eye morphogenesis” is a process in which the generation of anatomical structures of the eye happens and unifies (GO: 0048592).^[33]^

Furthermore, “visual perception” is a chain of incidents, which are essential for an organism to capture a visual stimulus, turn it out into a molecular signal, and describe and identify the signal. Signals are detected in the photon form and are converted to an image form (GO: 0007601). The definition of “photoreceptor cell differentiation” in the GO database is the specialization of formation of a photoreceptor, a cell that is responsive to electromagnetic ray, especially visible light (GO: 0001754). Drosophila melanogaster is an example of this procedure.^[33]^ “Camera-type eye development” (GO: 0043010) is a biological process, which its specific outcome is the progression of the camera-type eye over time, from its formation to a mature structure. The camera-type eye is an organ of sight that receives light through an aperture and focuses it through a lens, projecting it on a photoreceptor field.^[33]^

**Figure 3:**
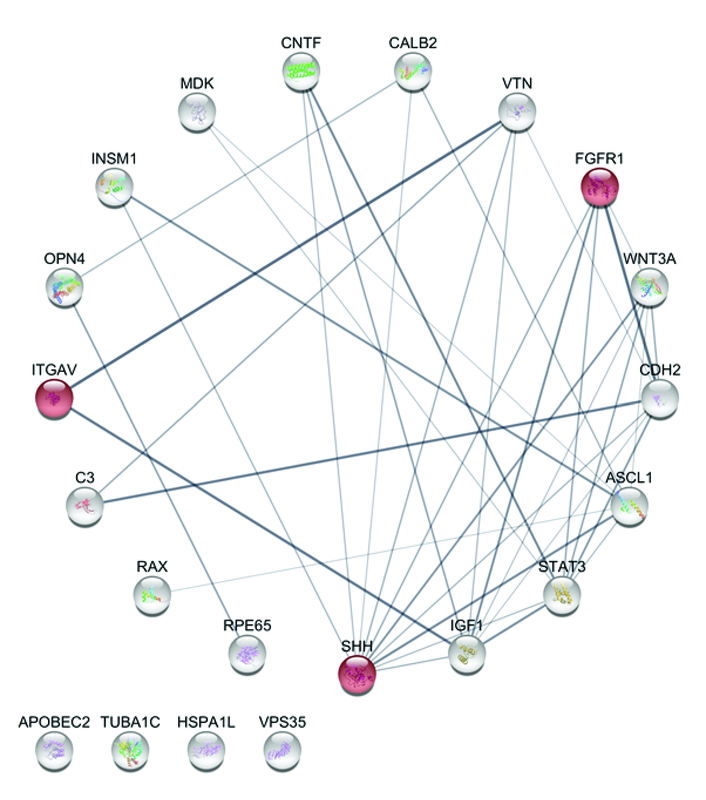
Angiogenesis involved proteins in retina regeneration, presented in a degree-sorted circular layout of the PPI network performed by STRING App in Cytoscape 3.7.2. Each circle provides a schematic drawing of protein structure. The colors are set randomly, and the connection line's thickness illustrates the relation of power. Also, a thicker line presents much more evidence and documents to approve the connectivity.

Angiogenesis is another crucial process that can lead to prosperous tissue fabrication. In fact, blood vessel formation is called angiogenesis when new vessels emerge from the proliferation of the pre-existing blood vessels. Based on evaluations, three proteins are involved in this process: SHH, FGFR1, and ITGAV. Figure 3 shows these proteins involved in the PPI network.

Regarding the most impressive protein-coding genes, which participate in those seven biological procedures based on GO analysis, that is, SHH, STAT3, FGFR1, OPN4, ITGAV, RAX, and RPE65, there are some interesting findings. SHH is able to induce angiogenesis, characterized by distinct large-diameter vessels and also augmented blood-flow recovery. *In-vitro*, SHH does not affect endothelial cell migration or proliferation; instead, it induces expression of two families of angiogenic cytokines, including all three vascular endothelial growth factor-1 (VEGF1) isoforms and angiopoietins-1 and -2 from the interstitial mesenchymal cells.^[35]^

Lack of FGF signaling in retinal pigment epithelium (RPE) during eye development strongly affects choroidal angiogenesis, including the absence of astrocytes, which are responsible for VEGF production. FGF-induced angiogenesis also requires activation of the VEGF system, while FGFs promote a strong angiogenic response.^[36]^ The product of this gene belongs to the integrin alpha chain family. Integrins are heterodimeric integral membrane proteins composed of an alpha subunit as well as a beta subunit that function in cell surface adhesion and signaling.^[37]^ However, the protein encoded by RPE65 is a component of the vitamin A visual cycle of the retina, which supplies the 11-cis retinal chromophore of the photoreceptors' opsin visual pigments. It performs the essential enzymatic isomerization step in the synthesis of the 11-cis retina. Mutations in this gene are associated with early-onset severe blinding disorders, such as Leber congenital amaurosis.^[38]^

Opsins are members of the guanine nucleotide-binding protein (G protein)-coupled receptor superfamily.^[39]^ OPN4 encodes a photoreceptive opsin protein that is expressed within the ganglion and amacrine cell layers of the retina. The protein functions as a sensory photopigment and may also have photoisomerase activity. Furthermore, RAX encodes a homeobox-containing transcription factor that functions in eye development.^[40]^ RAX is expressed early in the eye primordia and is required for retinal cell fate determination and regulates stem cell proliferation. Mutations in this gene have been reported in patients with defects in ocular development, including microphthalmia, anophthalmia, and coloboma.

Therefore, based on the extracted data from Table 2, SHH is accounted for eye development, camera-type eye development, and angiogenesis, whereas STAT3 is dedicated to eye development, photoreceptor cell differentiation, and eye morphogenesis. FGFR1 and ITGAV are also only involved in angiogenesis. OPN4 plays a great role in detecting visible lights and visual perception, while RAX is active in eye development, visual perception, and camera-type eye development. Finally, RPE65 is an impressive protein in all mentioned biological processes except angiogenesis.

Overall, explained biological processes and participated protein-coding genes must be considered in retina tissue fabrication. To fabricate artificial tissues or tissue regeneration, it is needed to understand the effective mechanisms to utilize them in an appropriate trend. Applying these proteins as growth factors may help in retinal tissue engineering. The results of this study positively correlate with earlier published reports. In fact, a variety of proteins have been shown to play a role in the retina development and regeneration process. The salient examples of these proteins are small peptide growth factors, SHH, taurine, epidermal growth factor (EGF), and FGF.^[41,42,43]^ RPE65, meanwhile, is considered as a strong marker for differentiation of bone marrow-derived stem cells (BMSC) into RPE.^[44,45,46]^ Furthermore, in the last decade, STAT3 was introduced as a recently recognized regulator of RPE survival. In addition, proliferation and visual cycle maintenance are functional roles of STAT3.^[47]^

In summary, due to the importance of retinal disorders and the growing need for tissue engineering in this field, *in-silico* studies are very useful to predict the general condition. This would lighten up the path and lead us to the right answer in an inexpensive way. In order to find out the most effective growth factors in retinal tissue engineering, an *in-silico* study was performed. This study demonstrates the importance and preview of the 21 proteins that play different roles in retinal regeneration.

According to each protein's biological function and role in different paths, selecting the correct ones is possible through the information that the network provides. Eye development, detection of visible lights, visual perception, photoreceptor cell differentiation, camera-type eye development, eye morphogenesis, and angiogenesis are the major biological processes in retinal regeneration. Based on GO analysis, each biological process has the most effective proteins in retinal regeneration, that is, SHH, STAT3, FGFR1, OPN4, ITGAV, RAX, and RPE65. In addition, based on degree index centrality analysis, the effectiveness of each protein on regeneration process was identified. In this regard, SHH, IGF1, STAT3, and ASCL1 are the proteins, which have the greatest influence on retinal regeneration. Based on these perspectives and nodes with the highest degree in the network, as well as GO analysis results, it is logical to focus on SHH, STAT3, and RPE65 in retinal tissue engineering.

##  Financial Support and Sponsorship 

This study has been funded and supported by Tehran University of Medical Sciences (TUMS); Grant no. 46271.

##  Conflicts of Interest

The authors declare that they have no conflict of interest.
